# Supplementary material for: Dog visits in nursing homes – increase complexity or keep it simple? A randomised controlled study
Source: PLoS One. 2021 May 26;16(5):e0251571. doi: 10.1371/journal.pone.0251571 (PMC8153477; doi:10.1371/journal.pone.0251571)
Supplement: S2 File — (PDF) [file pone.0251571.s002.pdf]

Protocol – Optimal dog visits (Optimale Hundebesøg)

Background

Visitor dogs in nursing homes are becoming widespread and accepted as a valuable intervention performed mainly by voluntary dog owners. The dogs are used as entertainment, company, for physical contact, interactions and probably provide a connection to the daily life and may remind the elderly of past times. A huge effort is already made to make dog contact available in Danish nursing homes, and we aim to produce results that will optimize the use of animal contact as a non-pharmacological intervention.

The scientific study of our relationship with companion and therapy animals is a growing field and in the recent years, some studies have been done on the nursing home population and in general, the findings are in support of using dogs as a therapeutic tool for this target group (Bernabei et al., 2013; Virués-Ortega et al., 2012).

We had the opportunity to perform an interdisciplinary study in 2011-2013, financed by TrygFonden, where we used ethological methods to observe the immediate response to dog visits and psychiatric instruments to test for effects on mental health, comparing dog visits with visits accompanied by either a welfare robot (PARO) or a soft toy cat. This study is one of the few large and well-designed investigations in this research field, and it enabled us to compare the response to a live dog, which could respond with the full repertoire of a live animal, with a robot giving some feedback and a soft toy cat only providing the opportunity of tactile stimulation. Our study showed that the residents were more likely to touch and interact with the dog and the interactive robot compared to the toy cat, which indicates, that the interaction and the feedback is important for the response. Furthermore, we found that the response depended on the cognitive functional level of the residents. The more impaired the residents were, the more they focused on the animal, whereas the least impaired group directed their attention significantly more towards the accompanying person. We found no effect on the prevalence of depressive symptoms or on the progression of cognitive impairment (two papers by Thodberg et al., submitted).

There have only been a few other comparable studies on the immediate response to dog visits in a nursing home setting. Kramer et al. (2009) found the same level of attention towards a live dog and a robotic dog (AIBO) measured as frequency and duration of looks, touch and talking; however, the study included only female residents, who received three visits in total from a person alone, a person with a live dog and a person with a robot dog (AIBO). Bernstein et al. (2000) compared a dog intervention with other activities in a study of 33 nursing home residents and found that the participants engaged in more physical contact and had longer conversations during dog visits compared to visits without dog. These findings are in line with results from our larger-scale study, underlining that feedback and interaction with a live animal or an interactive robot stimulates a response.

A larger number of studies have focused on more general or long-term effects of contact to dogs, especially the effect on the occurrence of depressive symptoms but, like in our study, none have found a significant reduction in depressive symptoms that could be attributed directly to dog visits (eg. Lutwack-Bloom et al., 2005; Zisselman et al., 1996). Only one relatively small study by Le Roux and Kemp (2009) found a significant reduction in the prevalence of depression. The dog contact in this study was offered to groups of nursing home residents instead of individually, and therefore the effect could also be due to social interaction with other residents during the visit.

Neither our study, nor previous studies have found an effect of dog visits on the cognitive capacity of nursing home residents; only Moretti et al. (2010) found such a tendency after six weeks of weekly dog group visits to moderately impaired nursing home residents.

Moreover, whereas long-term gains may be an unrealistic goal for populations who suffer from conditions causing permanent or even progressive loss of abilities, dog visiting programmes seem able to sweeten up the everyday life of nursing home residents (e.g., Banks & Banks, 2002; Lutwack-Bloom et al., 2005). This effect is worthwhile in itself, but will be masked in measures of competence level and pre- to post-intervention comparisons. Instead we need to monitor, during the visiting program, aspects of the residents’ day-to-day well-being and quality of life, such as immediate mood, daily social life, interactions with staff and fellow residents and, if possible, the roles that the visiting program takes in these.

Thus, there is need for more structured studies, where potential influential factors are investigated or controlled for. We have found that the interaction with the visiting animal is important for the response, and that the degree of cognitive impairment affects how the human-animal dyad is responded to. Now, we are ready for the next step, in which we aim to investigate whether enhancing the activity during a dog visit, providing the possibility for a more intense contact can induce an even stronger response, both during the visit, but also between visits and in the longer term. We learned in our previous project that impairment level affected the immediate response, and we would expect that residents with different cognitive abilities will respond differently to dog visits with different activity levels – an aspect that we now aim to include as well. Dog visits with a high activity level will be compared to an intervention without dog contact, but with the same level of activity. The knowledge from this new project will enable us to provide guidelines for optimal interventions with dogs for nursing home residents with different abilities and levels of cognitive impairment.

For a dog-assisted intervention to be successful it is important that the dogs behave in an optimal way that promotes the activity, and that the welfare of the dog is carefully monitored (e.g. Fine et al., 2010). However, not very many studies have examined this (Haubenhofer and Kirchengast, 2007; Glenk et al., 2013), and as we expect that the type of intervention will affect dog behaviour and welfare, we have included observations of the dogs in this study.

In this study, we will meet the highest scientific standards in terms of a) using random assignment to treatment and control groups, b) strict focus on standardisation at all possible levels and c) working in a multidisciplinary team. By including different fields of expertise it is possible to collect data on immediate behavioural and psychological responses as well as long-term psychiatric measures.

2. Aim and hypotheses

The aim of this project is to study

i) whether providing increased intensity of dog contact during dog visits in nursing homes affects the immediate response to and the general effect of the visits

ii) whether the level of cognitive impairment affects the response and benefit of dog visits

iii) whether more active dog visits have the same effect on nursing home residents with different levels of cognitive impairment

iv) how a visiting programme’s day-to-day effects on residents’ quality of life can be monitored

Our hypotheses are:

- that visits providing more intense dog contact will give a stronger response, both immediate and long-term, than visits with less intensity
- that residents with a high degree of cognitive impairment will respond more, both immediate and long-term, to visits with dogs than to visits without dogs
- that residents with a high degree of cognitive impairment will respond differently to visits with different activity levels
- that dog visits have positive effects on the residents’ daily life between visits

3. Relevance

It is highly relevant and needed to find and optimize non-pharmacological treatments and relevant activities for the increasing population of nursing home residents with disabilities such as dementia and related problems.

Furthermore, the results of this study will give support to the volunteers performing dog visits, and enable them and the staff to give optimal dog-assisted intervention to nursing home residents.

4. Material and Methods

4.1.Study design:

The study is a stratified randomised controlled study, where the participants are randomly allocated to receive one of three different visit types; Dog – low activity (DL), Dog – high activity (DH) and No Dog high activity (NDH). Before the random allocation the participants are divided into two different categories depending on their cognitive functional level. The combination of the three visit types and the two functional levels results in six treatment groups (Fig 1).

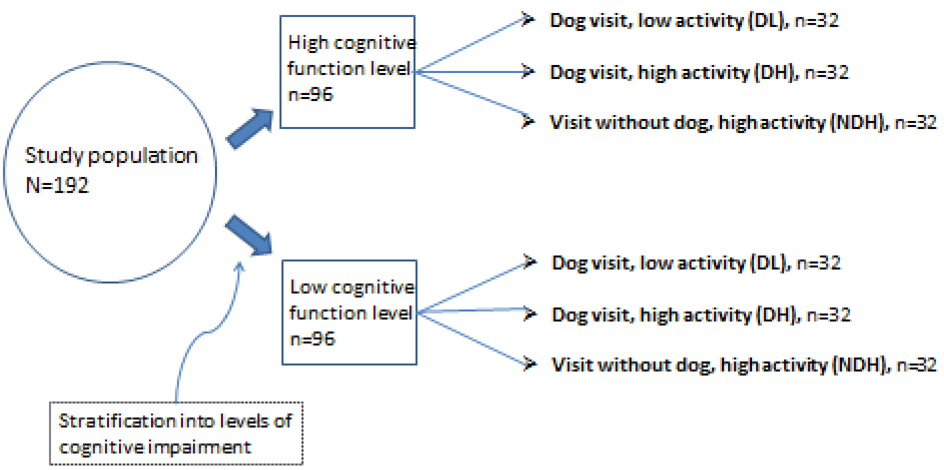

Figure 1: Study design

The study will be performed in six nursing homes in Denmark. We aim at having 32 participants in each nursing home and to collect data from a total of 192 nursing home residents. The study period in each nursing home will last eight weeks with two visits per week in the intermediate six weeks. The total data collection period will last approximately three years.

In each nursing home, two persons will perform the visits, and the visits conducted by these visitors will be balanced according to visit type and level of cognitive impairment of the receiver. In the individual nursing home, each visitor will work with the same dog throughout the experimental period.

After informed consent has been given, the participants are screened for cognitive functional level, using the Mini Mental State Examination scale (MMSE) and divided into cognitive impairment level 1 or 2 and subsequently randomly assigned to one of the three visit types. See section 4.5.2.1 for more information.

4.2. Inclusion and exclusion criteria:

Residents suffering from dog allergy or fear of dogs are excluded from the study. Furthermore, the participants must be able to sit up during the visits to make the experimental treatments comparable.

The participants must have a minimum age of 65 years. Finally, if we, during the screening procedure, become aware of health problems that make participation unadvisable, the residents are excluded.

All residents in the selected nursing homes that fulfil the above criteria will be given the opportunity to take part in the study.

4.3. Subdivision of participants into two cognitive levels:

Before the participants are allocated to the three visit types they are divided into either

Impairment level 1: MMSE >15, or

Impairment level 2: MMSE ≤ 15

4.4. Description of the visit types:

Each participant will be scheduled for 12 bi-weekly visits between 9 am and 4 pm, on either Mondays and Wednesdays or Tuesdays and Thursdays for six weeks. The residents will meet the same visitor and one of two observers in each visit. All visits will last 10 minutes and take place within the home of the resident. The conversation with the handler is not to be orchestrated, and it is

important that the handler and the dog, when present, contribute to a good atmosphere. If a dog is part of the treatment, it is fitted with heart rate measuring equipment before onset of the visit.

During the visit, the observer places himself/herself in the background and only joins the conversation if it feels naturally.

4.4.1 Treatment "Dog – low activity" (DL)

The visitor positions himself/herself close by the resident. The dog is on leash and within reach for at least 80% of the time, unless the resident clearly shows now interest at all or disapproves of contact to the dog. In the first visits, the dog will be introduced and the resident encouraged to make contact. The contact to the dog will not include play and training.

4.4.2 Treatment "Dog - high activity" (DH)

The same setup as with DL, but the resident is asked and encouraged to interact specifically and focused with the dog. The residents will be presented to a range of activities (brushing; making the dog sit and give a paw; playing a game with dog (e.g. hide and seek for treats; give the dog treats etc). The visitor will assist the resident performing these activities, and the time spent in each activity is registered.

4.4.3 Treatment "No dog - high activity" (NDH)

The visitor positions himself/herself close by the resident. The resident is encouraged to take part in an activity (simple game, singing a song; listen to music; loud reading etc.). The visitor will assist the resident performing these activities, and the time spent in each activity is registered.

After the second visit, all participants receive a picture of herself/himself together with the dog (DL, DH) or the visiting person (NDH).

4.5. Registrations:

4.5.1. Baseline measures

We collect a list of baseline information about the participating residents. These parameters have possible effects on the outcome and will therefore be included in the statistical model and used to describe the study population.

The baseline measures comprise:

- Age
- Gender
- Diagnoses
- Handicaps
- The use of extra medicine (PN)
- Family relations and other visits
- Education and previous occupation
- Dog allergy (these residents cannot be included in the study)
- Fear of dogs (these residents cannot be included in the study)
- Relationship and experience with dogs and other animals

4.5.2. Before, after and in between visits

4.5.2.1 Psychiatric measures before and after the 6-week visit period

The participants will be interviewed by a project nurse in the week before and after the six weeks with biweekly visits and scored on three psychiatric scales, all scored through interview and observation. The following instruments will be used: The Mini-Mental State Examination (MMSE) which gives information about the participants' cognitive state and possible cognitive malfunctions; The Gottfries-Bråne-Steen scale (GBS) that gives an evaluation of handicaps, language, psychiatric

symptoms, average daily living function and behaviour of the participants; and The Geriatric Depression Scale (GDS) that screens for depressive symptoms in elderly.

The instruments chosen are validated for this population and cover relevant and important aspects and general issues. Some of the measures will also build on information from the nursing home staff. The nurses will be "blind" with regard to the visit treatment that the residents will receive or have received.

4.5.2.2. Psychological measures between visits

Between visits, the members of the staff, who are appointed as contact persons for participants, will be asked to assess the residents' mood, how he or she socialises with the staff and fellow residents and other indicators of well-being and quality of life.

We shall develop a tool specifically aimed at this assessment, using experiences from our previous study of visiting dogs in nursing homes. In that study, we did not collect data between visits, but the staff reported several examples of what they thought were effects of the experimental visits. We shall conduct interviews with at least three members of staff from each of the four nursing homes that took part in that study, identify responses that can be used as an indicator of an effect and combine these with relevant items from existing scales for measuring mood, interaction and Quality of Life of elderly and nursing home residents, such as NPI (NH) (Wood et al., 2000), POMS (Nyenhuis et al., 1999), PAS (Rosen et al., 1994), SAPS (Hawthorne et al., 2006; Hawthorne, 2008) and DEMQOL (Smith et al., 2005). The bruttolist of items thus derived will be evaluated with a subset of participants during the second year of the study and form the basis for a condensation to maximum 15 items for the final instrument.

4.5.3. During visits.

4.5.3.1. Physiological measures to be measured during visits

<u>Dogs</u>

- Heart rate measured by the application of a Polar heart rate monitor

<u>Humans</u>

If we are able to find extra funding we will include measures of heart rate derived from video recordings of the participants during visits. By the use of video enhancement technique, the heart rate can be monitored from video recordings of the face of the residents using the software MATLAB. Data on heart rate are collected once a week in 6 of the 12 visits. If we receive funding we will apply permission for retrieving these data in an additional application.

4.5.3.2. Behavioural registrations during visits

The behaviour will be registered by direct observation of behavioural elements from a predefined list (an ethogram). In dog visits we register the residents' focus on and contact to the dog measured as tactile contact, eye contact, verbal communication. In all visits, we will register the residents' focus on the activity presented, conversation and tactile contact with the visitor as well as gesticulation.

The dogs' behaviour will also be registered into categories of distance and contact seeking behaviour as well as the general activity.

We will use the sampling method *all occurrence* that provides true frequencies and durations of the behavioural elements.

During visits with a dog, the dog will be video recorded.

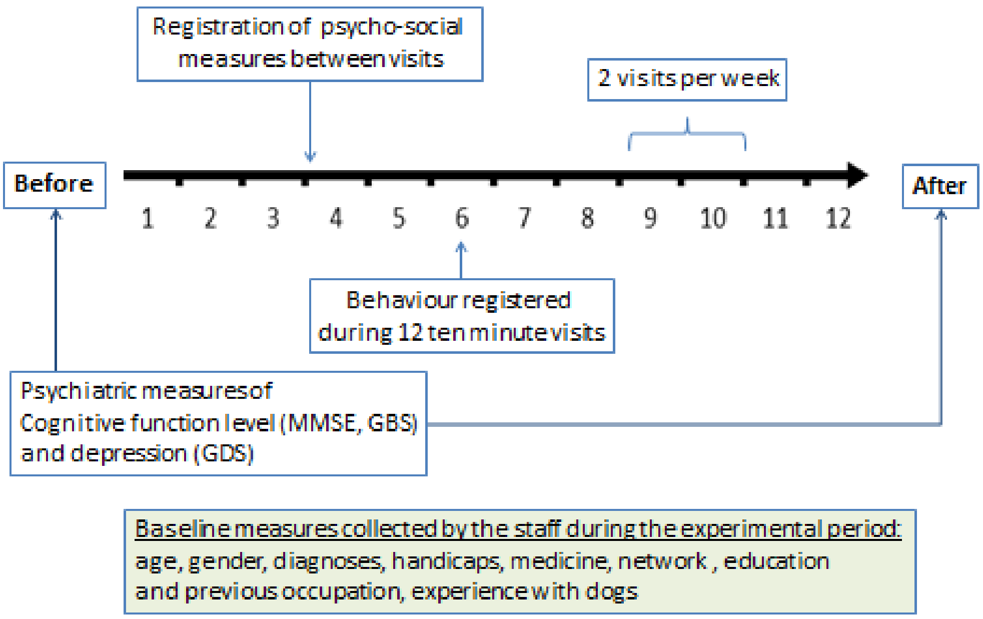

Figure 2. Overview of registrations. The timeline covers the 6-week visit period and the numbers indicate the 12 individual visits.

5. Dogs

The dogs used in the study will be approved by "TrygFondens besøgshunde" (www.besoegshunde.dk).

6. Statistical analysis

The design of the study is a stratified and randomised controlled design, where the participants are allocated to three treatments after being categorised according to cognitive functional level. We expect 192 participants in six nursing homes.

The data will be analysed in a linear mixed model or a general linear mixed model depending on the distribution of the data. Non-parametric statistics will be used if necessary.

The measures of effect include individual and repeated measures of immediate behavioural responses to the treatment sessions, as well as short- and long-term psychological measures. The systematic effects will be the different treatment and the cognitive impairment level and other continuous and categorical factors will be included as well, e.g. the gender and age of the residents, and the identity of visiting persons and dogs. The different nursing homes will be modelled as random effects.

All personal data will be treated confidentially and to ensure anonymity, each participant will be assigned a number, which will be used during data analysis and presentation of the results.

The size of the study is comparable to our previous study working with similar effect measures, and therefore we strongly believe that the power in this study is sufficient. Compared to studies in similar settings, this study is very large.

7. Medical and ethical considerations – secondary effects, risks and disadvantages

The three visit situations are expected to be positive events, and are non-invasive in nature with no known adverse effects.

The risks for the participant include unforeseen allergic reactions and the transmission of disease or infection from the dog. In general, the risk of transmission of zoonoses from dogs to humans is very low (Brodie et al., 2002; Yamauchi and Pipkin, 2008), even though a large proportion of dogs probably will be infected (Lefebvre et al., 2006). However, in a study of immune-compromised people, no additional risk by interacting with pets compared to interaction with persons was found (Hemsworth and Pizer, 2006). However, all dogs will go through a clinical examination before the onset of the visit period and residents with known allergy are excluded from the study.

We expect that up to 80% of the nursing home residents have some level of cognitive impairment and a subgroup of these must be expected to be incompentent (inhabile). We wish to include incompetent persons in the study as they are a subset of the primary target group for dog visits in nursing homes. Especially people suffering from different types of dementia are of particular interest, as they often have problems with communication and therefore are difficult to ask

questions about how they experience dog visits. By using behavioural observations we can describe their response to the different visit types even if they do not communicate verbally.

Especially, there is a need to study non-pharmacological treatment and activity options for nursing home residents – especially for the subgroup suffering from cognitive impairment. The number of non-pharmacological treatment offers without side effects is very limited at the moment. This project will provide highly needed scientific evidence and lead to a development in the animal-assisted treatments and activities for nursing home residents. The project could not, with the same utility, be accomplished by using only competent (habile og myndige) persons (cf. Komiteloven § 19).

The study does not include experiments with medicine and will generate knowledge that can result in advantages for the nursing home residents, including persons suffering from dementia. This could for instance be achieved through more targeted use of dog visits in the future. Furthermore, taking part in the study will presumably be a positive experience for the individual resident and we assess that only minimal risks and discomfort are connected with taking part in the study (cf. Komiteloven § 19).

There can be ethical considerations about introducing a positive intervention in a limited period of time, however, the six nursing homes that will be included will be able to acquire dog visits from *TrygFonden Besøgshunde* after the end of the study period.

The participants that receive the control visits without the dog could perhaps be disappointed about this. However, as this visit type also includes an activity, we assume that this will not be problematic. Since not all participants will receive dog visits we wish to remove the word “dog” (hund) from the title of the information material, in order not to disappoint the expectations of the participants. This is based on our experience from our last project, where the participants had visits from a person accompanied either by a dog, a robot seal or a soft toy cat. The full title of the project is given in the information material in the section describing the funding of the study.

8. Information and declaration of consent

Before the start of each study period, the management at the nursing home is contacted and an information meeting for the staff is scheduled (two months before).

One to two months before the project period starts, written information material is sent to the potential participants. Subsequently, and no later than 2 weeks before the data collection starts, each potential participant receives the information orally at a meeting with a nurse from the project group in their own home, and signs a declaration of informed consent, if he or she wants to take part in the study. If the potential participant needs more time to decide whether he or she would like to take part in the study, another meeting is scheduled within a week from the first meeting. If the potential participant wants a member of the staff or a relative to be present during such a meeting, this is arranged.

If the resident is disqualified/incompetent (inhabil) and not able to make decisions on her/his own, a relative, his/her own doctor or the health inspector from the Danish Health and Medicines Authority (embedslægefunktionen) is provided with written information material one to two months before the start of the project period. Subsequently, and no later than 2 weeks before the data collection starts, this person will receive the information orally from a nurse from the project group. If this person assesses that the potential participant can participate, he or she can sign a special declaration of informed consent on their behalf. If this person needs more time to decide whether the potential participant can participate, another meeting is scheduled within a week from the first meeting. The decision as to whether a potential participant is incompetent is taken by the manager/leader of the nursing home. By signing the declaration, the resident or the alternate confirms the participation in the study. The participants or their alternates can at any time withdraw their consent.

In the information material it is clearly stated what it implies to take part in the study. The participants will not receive any form of fee in connection to their participation in the project.

9. Time schedule and milestones

The project will run from January 1st 2015 until December 31st 2018. The milestones and their timing are shown in Table 1.

Table 1. Time schedule of the project

**Milestones**	**2015**	**2016**	**2017**	**2018**
Permission retrieval<br>Preparation of information material<br>Adjustment of protocol				
Pilot study/pilot visits				
Interviews with staff from previous experimental nursing home to gain input for the between visit evaluation tool.				
Contact to nursing homes<br>Contact to dog owners<br>Obtaining informed consent				
Data collection				
Analysis of video recordings<br>Statistical analysis				
Publication				
Education material				

10. Funding of the project:

The applicant is the initiator of the project, which is financed by TrygFonden by 4.500.000 DKK (incl. overhead). This sum will cover the expenses for salary and mileage expenses and the purchase of materials. The money will be paid at the end of each year after TrygFonden has received an account. The group of scientists involved in the project are impartial in relation to TrygFonden, who has no influence on how we conduct the experiment.

11. Collaborators and organisation

The project involves partners from the Department of Animal Science and Department of Clinical Medicine, Aarhus University and Department of communication and Psychology, Aalborg University.

Department of Animal Science, Science and Technology, Aarhus University:

Senior scientist, PhD, Karen Thodberg (Applicant): Project leader and responsible for the project management and collection of immediate behavioural and physiological data. Karen has extensive experience with statistical data analysis.

Scientist Janne Winther Christensen: Co-responsible for data collection during the experiments. Janne is highly experienced in data collection and human-animal interactions.

Department of Clinical Medicine, Aarhus University

Professor Dr. Med. Poul Videbech will be in charge of data collection within the psychiatric field and will, (together with Karen Thodberg), lead the work of the project nurses.

One or two project nurses will be employed in the project to perform the collection of psychiatric data; help with providing information to the potential participant; and obtaining informed consent.

Department of communication and Psychology

Associate professor, PhD, Tia G. B. Hansen will be in charge of collecting psycho-social data from the nursing home staff regarding their observations of the effects on the residents.

Due to the interdisciplinary character of the study, the responsibility of each professional field (the immediate behavioural/physiological, psychological and the psychiatric part) is managed by Karen Thodberg, Poul Videbech and Tia G.B. Hansen, respectively.

12. Strategy for publication and communication

The results will be published in international peer reviewed papers, at international conferences and in Danish popular magazines. Both positive, negative and inconclusive results will be reported. We expect that the project will receive media attention, and we are prepared for this.

13. References

Bank, M.R., Banks., W.A., 2002. The effect of animal-assisted therapy on loneliness in an elderly population in long-term care facilities. J. Gerontology: Medical sciences. 57a: M428-M432.

Bernabei, V., De Ronchi, D., La Ferla, T., Moretti, F., Tonelli, L., Ferrari, B., Forlani, M., 2013. Animal Assisted interventions for elderly patients affected by dementia or psychiatric disorders: A review. J. Psych. Research, 47: 762-773.

Bernstein, P. L., Friedmann, E., Malaspina, A., 2000. Animal assisted therapy enhances resident social interaction and initiation in long-term care facilities. Anthrozoös, 13: 213-224

Brodie, A.J., Biley, F.C., Shewring, M., 2002. An exploration of the risks associated with using pet therapy in healthcare settings. Journal of Clinical Nursing., 11: 444-456.

Fine, A H., 2010. Handbook on animal assisted therapy .theoretical foundations and guidelines for practice, Academic Press.

Glenk, LM, Kothgassner, O.D., Stetina, B.U., Palme, R., Kepplinger, B., Baran, H., 2013. Therapy dogs’ salivary cortisol levels vary during animal-assisted intervention. Animal Welfare, 22: 369-378.

Haubenhofer, D. K., Kirchengast, S., 2007. Dog handlers' and dogs' emotional and cortisol secretion responses associated with animal-assisted therapy sessions. Society and animals, 15: 127-150.

Hawthorne, G. (2008). Suggested revisions to the SAPS. Retrieved November 26 from http://www.dementia-assessment.com.au/symptoms/SAPS.pdf

Hawthorne, G., Sansoni, J., Hayes, L., Marosszeky, N., & Sansoni, E. (2006). Measuring patient satisfaction with incontinence treatment. Report, Centre for Health Service Development, University of Wollongong and the Department of Psychiatry, University of Melbourne.

Hemsworth, S.; Pizer, B., 2006. Pet ownership in immunocompromised children-A review of the literature and survey of existing guidelines. European Journal of Oncology Nursing, 10: 117-127.

Kramer, S.C., Friedmann, E., Bernstein, P.L., 2009. Comparison of the effect of human interaction, animal-assisted therapy, and AIBO-assisted therapy on long-term care residents with dementia. Anthrozoös, 22: 43-57.

Lefebvre, S. L., Golab, G. C., Christensen, E., Castrodale, L., Aureden, K., Bialachowski, A., Gumley, N., Robinson, J., Peregrine, A., Benoit, M., Card, M. L., Van Horne, L., Weese, J. S., 2008. Guidelines for animal-assisted interventions in health care facilities. American Journal of Infection Control, 36: 78-85.

Le Roux, M.C., Kemp, R., 2009. Effect of a companion dog on depression and anxiety levels of elderly residents in a long-term care facility. Psychogeriatrics, 9: 23-26.

Lutwack-Bloom, P., Wijewickrama, R., Smith, B., 2005. Effects of pets versus people visits with nursing home residents. Journal of gerontological social work, 44: 137-159 et al., 2005;

Moretti, F., De Ronchi, D., Bernabei, V., Marchetti, L., Ferrari, B., Forlani, C., Negretti, F., Sacchetti, C., Atti, A.R., 2011. Pet therapy in elderly patient with mental illness. Psychoreriatrics., 11: 125-129.

Nyenhuis, D. L., Yamamoto, C., Luchetta, T., Terrien, A., & Parmentier, A. (1999). Adult and geriatric normative data and validation of the profile of mood states. Journal of Clinical Psychology, 55(1), 79-86.

Rosen, J., Burgio, L., Kollar, M., Cain, M., Allison, M., Fogleman, M., . . . Zubenko, G. S. (1994). The Pittsburgh Agitation Scale: A user-friendly instrument for rating agitation in dementia patients. The American journal of geriatric psychiatry : official journal of the American Association for Geriatric Psychiatry, 2(1), 52-59.

Smith, S. C., Lamping, D. L., Banerjee, S., Harwood, R., Foley, B., Smith, P., . . . Knapp, M. (2005). Measurement of health-related quality of life for people with dementia: development of a new instrument (DEMQOL) and an evaluation of current methodology. Health technology assessment, 9(10), 1-93.

Thodberg, K., Sørensen, L. U., Videbech, P. B., Poulsen, P.H., Houbak, B., Damgaard, V., Keseler, I., Edwards, D., Christensen, J. W. Immediate behavioral responses of nursing home residents to visits with a dog, a robot seal or a soft toy cat. Submitted to Anthrozoös

Thodberg, K., Uhrskov, L., Christensen, J. W., Edwards, D., Poulsen, P., Houbak, B., Damgaard, V. J., Keseler, I., Videbech, P., 2013b.The effects of biweekly visits accompanied by either a dog, a robot seal or a soft toy cat on sleep patterns and mental health of nursing home residents. Submitted to The American Journal of Geriatric Psychiatry.

Virués-Ortega, J., Pastor-Barriuso, R., Castellote, J, M., Poblacion, A., de Pedro-Cuesta, J., 2012. Effect of animal-assisted therapy on the psychological and functional status of elderly populations and patients with psychiatric disorders: a meta analysis. Health psychology review, 6: 197-221.

Wood, S., Cummings, J. L., Hsu, M.-A., Barclay, T., Wheatley, M. V., Yarema, K. T., & Schnelle, J. F. (2000). The use of the neuropsychiatric inventory in nursing home residents: Characterization and measurement. The American Journal of Geriatric Psychiatry, 8(1), 75-83. doi: http://dx.doi.org/10.1097/00019442-200002000-00010

Yamauchi, T., Pipkin, E., 2008. Six year experience with animal assisted therapy in a children's hospital : Is there patient risk? American JOuranl of Infection Control, 11-113, p E117.

Zisselman, M.H., Rovner, B.W., Shmuely, Y., Ferrie, P., 1996. A pet therapy intervention with geriatric psychiatry inpatients. The american journal of occupational therapy, 50: 47-51.

14. Strategy for literature search:

The literature on visiting dogs is found within many different research fields (psychiatry, psychology and ethology). We primarily searched using "Web Of Science" with the following search words: animal assisted therapy; pet therapy; visiting dogs, in combination with: nursing home resident; dementia. Furthermore, new references, which were not uncovered by this primary search, were found in the resulting papers.
