# Supplementary material for: Dog visits in nursing homes – increase complexity or keep it simple? A randomised controlled study
Source: PLoS One. 2021 May 26;16(5):e0251571. doi: 10.1371/journal.pone.0251571 (PMC8153477; doi:10.1371/journal.pone.0251571)
Supplement: Attachment — Submitted filename: renamed_1bcf7.docx [file pone.0251571.s003.docx]

**COMMENTS – Manuscript PONE-D-20-34638**

Title: “Dog visits in nursing homes – increase complexity or keep it simple?: A randomised controlled study”

**Important note: This review pertains only to ‘statistical aspects’ of the study and so ‘clinical aspects’ [like medical importance, relevance of the study, ‘clinical significance and implication(s)’ of the whole study, etc.] are to be evaluated [should be assessed] separately/independently. Further please note that any ‘statistical review’ is generally done under the assumption that (such) study specific methodological [as well as execution] issues are perfectly taken care of by the investigator(s). This review is not an exception to that and so does not cover clinical aspects {however, seldom comments are made only if those issues are intimately / scientifically related & intermingle with ‘statistical aspects’ of the study}. Agreed that ‘statistical methods’ are used as just tools here, however, they are vital part of methodology [and so should be given due importance].**

**COMMENTS:** Actually, your ABSTRACT is well drafted but assay type. Please note that it is preferable [refer to item 1b of CONSORT checklist 2010: Structured summary of trial design, methods, results, and conclusions] to divide the ABSTRACT with small sections like ‘Objective(s)’, ‘Methods’, ‘Results’, ‘Conclusions’, etc. which is an accepted practice of most of the good/standard journals [including this one]. It will definitely be more informative then, I guess, whatever the article type may be.

I agree with what Friedman et al. (2019) reported according to authors of this article that [lines 97-98] ‘no association between impairment level, and recorded behaviour during dog visits’. Important factor not taken care of here is ‘whether subject [dementia case] like the pets?’ In my experience, there are many people who do not like pets {I am not very sure about situation in Europe (though I had been there several times and stayed), but I am very much sure about situation in my country}. This factor could be a ‘confounder’ and needs to be adjusted for, [or may totally exclude such cases], in my opinion.

Because of the above and authors aimed to study whether enhancing the possibility for interaction with the dog during dog visits is beneficial for all [as said in lines 109-10], this review may be ‘biased’, I think. Although one of the exclusion criteria {line 144} is ‘fear of dogs’, I guess, not liking pets/dogs is not exactly same as ‘fear of dogs’. Mention in line 194/195 of the fact that ‘disapproved of contact with the animal’ implies there were at least few of this type.

Information given regarding estimation of ‘Sample size required’ (lines 164-6) is inadequate. Is it assumed that 12 (each) different activities listed in table 1 & 2 are of the same type/intensity? Is it correct to impute/substitute the values of the missed items with the median [of this item across participants]? In back-drop of ‘level of measurement’ {and/or type of variables} of ‘Behaviour variables’ [all measurements], ‘Non-parametric models’ are more sensible it seems. Many other things are analysed and discussed. Most of them appears alright [statistically], however, ‘clinical aspects’ [like medical importance, relevance of the study, ‘clinical significance and implication(s)’ of the whole study, etc.] are to be evaluated [should be assessed] separately/independently, I feel.
