# Supplementary material for: Dog visits in nursing homes – increase complexity or keep it simple? A randomised controlled study
Source: PLoS One. 2021 May 26;16(5):e0251571. doi: 10.1371/journal.pone.0251571 (PMC8153477; doi:10.1371/journal.pone.0251571)
Supplement: Attachment — Submitted filename: Response to reviewers_230421 (2).docx [file pone.0251571.s005.docx]

**PONE-D-20-34638R1
Dog visits in nursing homes – increase complexity or keep it simple? A randomised controlled study
PLOS ONE**

Question from the editor:

An outstanding issue is your statement concerning sample size that remains inadequate. It should include all the specific numerical details that will allow the reader to reproduce your calculation - rather than the current broad description ("behavioural data from our previous studies"

Answer:

I have revised the section about sample size and included more numerical details (lines: 163-173).

The line numbers refer to the manuscript version without track changes.

In Table 1, the number five “5” has been added in the line describing activity number five.

The manuscript with track changes is the same version as submitted in March, and include the track changes made back then as well as the present changes
